# The drug-survival of low-dose thioguanine in patients with inflammatory bowel disease: a retrospective observational study

**DOI:** 10.1177/17562848241228064

**Published:** 2024-02-20

**Authors:** Helena Gensmyr-Singer, Mårten Werner, Pontus Karling

**Affiliations:** Department of Public Health and Clinical Medicine, Umeå University, Umeå, Sweden; Department of Public Health and Clinical Medicine, Umeå University, Umeå, Sweden; Department of Public Health and Clincal Medicine, Umeå University, Umeå S-90811, Sweden

**Keywords:** azathioprine, calprotectin, Crohn’s disease, inflammatory bowel disease, mean corpuscular volume, mercaptopurine, thioguanine, thiopurine, ulcerative colitis

## Abstract

**Background:**

Thiopurines are commonly used to treat inflammatory bowel disease but withdrawal due to side effects are common. Thioguanine has been suggested to be better tolerated than conventional thiopurines.

**Objectives:**

We studied drug-survival of low dose of thioguanine in real-life clinical practice in comparison to conventional thiopurines.

**Design:**

Retrospective observational study.

**Methods:**

All patients born 1956 and later, and who at least once started thiopurine treatment between 2006 and 2022 were included. A medical chart review was performed that noted drug-survival for every thiopurine treatment attempt. The Mantel–Cox rank test was used to test differences in drug-survival for different thiopurines. Blood chemistry analysis and faecal calprotectin levels were registered for the first 5 years of treatment.

**Results:**

In the study population, there was 379 initiated thiopurine treatments (210 for Crohn’s disease and 169 for ulcerative colitis) in 307 patients with inflammatory bowel disease (IBD). Low-dose thioguanine (median dose 11 mg; 25–75th percentile 7–19 mg) had been initiated in 31 patients. Overall, when including all thiopurine attempts, thioguanine had the longest drug-survival [Mantel–Cox rank test: thioguanine *versus* azathioprine *p* = 0.014; thioguanine *versus* 6-mercaptopurine (6-MP) *p* < 0.001]. For second-line thiopurine treatment thioguanine had longer drug-survival than 6-MP (Mantel–Cox rank test: *p* = 0.006). At 60 months, 86% of the patients who started low-dose thioguanine were still on treatment compared to 42% of the patients who started 6-MP (*p* = 0.022). The median 6-thioguanine nucleotide levels in patients treated with thioguanine was 364 pmol/8 × 10^8^. Patients on thioguanine treatment showed significantly lower values of median mean corpuscular volume at follow-up than patients treated with azathioprine and 6-MP. Patients treated with 6-MP showed significantly lower levels of FC in the third year of treatment compared to patient treated with azathioprine (59 *versus* 109 µg/g; *p* = 0.023), but there was no significant difference in FC levels for thioguanine compared to azathioprine (50 *versus* 109 µg/g; *p* = 0.33).

**Conclusion:**

Treatment with a low dose of thioguanine is well-tolerated in patients with IBD and had a significantly higher drug-survival than conventional thiopurines.

## Plain language summary

Low-dose of the immunomodulator drug thioguanine are well tolerated by patients with inflammatory bowel disease

Thiopurines are commonly used to treat inflammatory bowel disease but it is common that patients end treatment due to side-effects. The thiopurine thioguanine has been suggested to be better tolerated than other thiopurines. We aimed to study if a low-dose of thioguanine had been tolerated better and used longer than other thiopurines in patients with inflammatory bowel disease at our clinic. In the study population there was 379 initiated thiopurine treatments in 307 patients with inflammatory bowel disease. Among those patients a low-dose thioguanine had been initiated in 31 patients. Overall, when including all thiopurine attempts, thioguanine had longest drug-survival of all thiopurines. For second line thiopurine treatment thioguanine had longer drug-survival than the thiopurine 6-mercaptopurine that are usually used as second line thiopurine treatment. At 60 months, 86% of the patients who started low dose thioguanine was still on treatment compared to 42% of the patients who started 6-mercaptopurine.There was a similar response on inflammatory markers the first five years from starting treatment with thioguanines compared to conventional used thiopurines. We conclude that treatment with a low-dose of thioguanine is well tolerated in patients with inflammatory bowel disease and have a significantly higher drug survival than conventional thiopurines.

## Introduction

Thiopurines are purine analogues that reduce cell proliferation and have immune modulating properties and are predominantly used in the treatment of leukaemia but in lower doses are also used for autoimmune inflammatory disorders.^1^ Since the 1960s, the thiopurines azathioprine, 6-mercaptopurine and thioguanine have been used to treat inflammatory bowel disease (IBD).^2^ Approximately 40% of the patients with Crohn’s disease (CD) and 20% of patients with ulcerative colitis (UC) will start thiopurine treatment within the first 5 years since diagnosis.^3^ Both azathioprine and 6-mercaptopurine are effective in the maintenance treatment of CD^4,5^ and UC.^6^ Thiopurines may also inhibit the production of auto antibodies for biologics when treating patients with IBD.^7,8^ Evidence for thioguanine in the treatment of IBD is of low quality and is based mostly on patients treated with experimental or rescue therapy.^9^ Currently, there are no randomized control trials that have compared thioguanine with placebo or other treatments for IBD. Approximately 15–40% of the patients treated with thiopurines experience side effects that lead to drug withdrawal or reduction of dosages.^10^ The side effects are either dose-dependent (i.e. nausea, myelotoxicity, hepatotoxicity) or dose-independent (i.e. malaise, fever, musculoskeletal pain, pancreatitis).^10,11^

**Figure 1.**
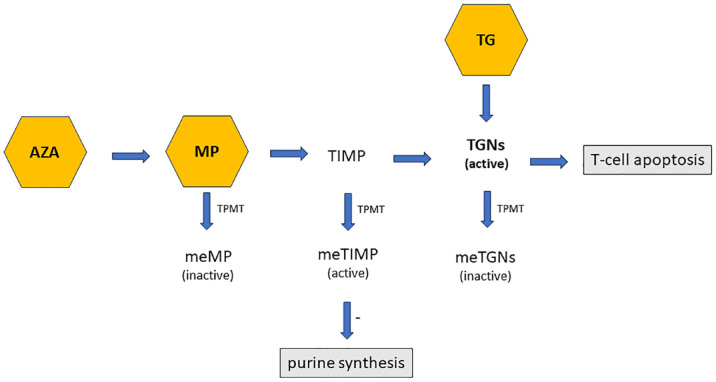
Thiopurine drug metabolism pathway. AZA, azathioprine; meMP, methyl-mercaptopurine; meTGNs, methyl-thioguanine nucleotides; MeTIMP, methyl-thioinosine 5′-monophosphate; MP, mercaptopurine; TG, thioguanine; TGNs, thioguanine nucleotides; TIMP, thioinosine 5′-monophosphate; TPMT, thiopurine methyltransferase.

In comparison to azathioprine and 6-mercaptopurine, thioguanine has the advantage to ‘skip’ some steps in the metabolism that are known to be associated with adverse events^12^ (Figure 1). For example, there is no formation of methyl-thioinosine 5′ monophosphate (MeTIMP) when using thioguanine treatment. Thioguanine was also suggested to be a more effective treatment and possibly have a more rapid onset of action than other thiopurines.^13^ In a recently published prospective study, 45% of the patients who were intolerant to conventional thiopurine were still in corticosteroid-free clinical remission at 12 months on thioguanine treatment.^14^ In addition, there are promising results for using thioguanine in patients naïve for thiopurines. In a study from the Netherlands, approximately half of the patients naïve to thiopurines who started thioguanine were free of steroid, biological agents and surgery after 1 year of treatment.^15^ Since 2022, thioguanine has been licenced in the Netherlands to be used for adult patients with IBD.^14^

At our clinic in Sweden, we have traditionally used lower doses of thioguanine in the treatment of IBD in patients who are intolerant to azathioprine and/or 6-mercaptopurine. In Sweden, thioguanine is available as 40 mg tablets, and at our clinic, we use split-doses of thioguanine therapy of ‘half tablets’ (20 mg) once or every second day. The present retrospective observational study compared the drug-survival of low-dose thioguanine with other thiopurine treatments overall and with 6-mercaptopurine for patients who failed or were intolerant to azathioprine.

## Methods

### Design

This is a retrospective observational study of patients that started treatment with thiopurines for IBD in clinical practice. The reporting of this study conforms to the Strengthening the Reporting of Observational Studies in Epidemiology (STROBE) statement.^16^

### Patients

Included patients were patients with endoscopically or imaging verified diagnosis of IBD and who were treated at the Department of Medicine at Norrlands University Hospital, Umeå, Sweden. The study was restricted to patients born in 1956 or later and who had been diagnosed with IBD after the year of 2006. Patients diagnosed before 2006 were excluded because complete data on drug prescription in the digital medical record system were not reliable until 2006. A patient had to be at least 18 years old when starting thiopurine treatment. An exclusion criterion was patients diagnosed or treated outside the catchment area.

### Medical chart review

A thorough medical chart review was conducted that focused on drug-survival for every thiopurine treatment that started after diagnosis and up until 31 December 2022. Drug-survival in our study was solely defined by continuation of treatment, and the patients were allowed to add other IBD treatments. For every start of thiopurine treatment, the type of thiopurine, time on treatment and reasons for discontinuation of treatment were noted. The median dosage of each thiopurine treatment was calculated. Second-line therapy was defined if a thiopurine treatment was initiated after failure or withdrawal of a first thiopurine drug. For the first 5 years median yearly values for haemoglobin, white blood cells count, mean corpuscular volume (MCV), white blood cells count, lymphocytes, neutrophils, C-reactive protein, albumin and faecal calprotectin were registered for every attempt of thiopurine treatment. Also registered were the maximal values of aspartate amino transferase (ASAT), alanine amino transferase (ALAT), alkaline phosphatase (ALP) and pancreatic amylase. The median levels of 6-thioguanine nucleotide (6-TGN) and the maximal value of MeTIMP for the first 5 years after starting treatment were also noted. The combinate use of tumour necrosis factor (TNF) inhibitors was registered for the first 5 years since start of treatment. At our hospital, the method use for faecal calprotectin analysis is the CALPRO^®^ Calprotectin ELISA Test (ALP).^17^ The quantitative analysis of thiopurine drug metabolites in whole blood was performed by the liquid chromatography-mass spectrometry (LC-MS/MS method).^18^

### Statistics

IBM SPSS Statistics version 28.1.1 was used for data analysis. The chi-square test and Fisher exact test were used to compare proportions. The Mann–Whitney *U-*test was used to compare continuous data that was not normally distributed. Kaplan–Meier curves were used to illustrate drug-survival and the Mantel–Cox rank test was used to calculated statistical differences in drug-survival. A *p*-value <0.05 was considered statistically significant. There were no corrections done for multiple testing.

## Results

### Baseline characteristics

Thiopurine treatment had been prescribed at least once in 64.1% of the patients with CD and in 32.1% of the patients with UC (*p* < 0.001). In the study population, there was 379 initiated thiopurine treatments (210 for CD and 169 for UC) in 308 patients with IBD. The median time from diagnosis to start of thiopurine treatment was significantly shorter for patients with CD than patients with UC (4 *versus* 16 months; *p* < 0.001). The median time on thiopurine treatment was significantly longer in patients with CD than those with UC (35 *versus* 28 months, *p* = 0.030), but there was no significant difference in drug-survival between patients with CD and UC using the Mantel–Cox log rank test (*p* = 0.618).

Patients who were treated with thioguanine were significantly older at diagnosis compared to patients who started other thiopurine treatments (Table 1). There were no differences in gender, type of diagnosis, Montreal classification, treatment at baseline and surgery before treatment between patients who were treated with thioguanine, and patients treated with other thiopurines.

**Table 1.** Baseline characteristics for patients treated with thioguanine and patients treated with other thiopurines.

Baseline characteristics	Patients who started thioguanine treatment (*N* = 31)	Patients who started other thiopurine treatment (*N* = 276)	*p*-Value
Median age when diagnosed; years (25th–75th percentile)	35 (24–45)	26 (20–36)	0.009
Median time from diagnosis to first thiopurine start; months (25th–75th percentile)	16 (2–39)	7 (2–25)	0.694
Median time on thiopurine treatment; months (25th–75th percentile)	34 (12–65)	32 (12–70)	0.925
Median observation time from diagnosis; months (25th–75th percentile)	87 (41–145)	75 (38–124)	0.374
Gender, % (*N*)			0.416
Women	48 (15)	41 (113)	
Men	52 (16)	59 (163)	
Ulcerative colitis, % (*n*)	42 (13)	46 (127)	0.651
Crohn’s disease, % (*n*)	58 (18)	54 (144)	0.651
Montreal classification age, % (*N*)			0.080
A1 (<17 years)	9 (1)	9 (54)	
A2 (17–40 years)	70 (19)	64 (196)	
A3 (>40)	21 (11)	17 (27)	
Montreal classification UC extension, % (*N*)			0.375
E1 (ulcerative proctitis)	8 (1)	5 (6)	
E2 (left-sided ulcerative colitis)	54 (7)	37 (46)	
E3 (extensive ulcerative colitis)	38 (5)	59 (75)	
Montreal classification Crohn location, % (*N*)			0.632
L1 (small intestine)	39 (7)	32 (48)	
L2 (colon)	50 (9)	48 (71)	
L3 (small intestine and colon)	11 (2)	20 (30)	
Montreal classification Crohn behaviour, % (*N*)			0.604
B1 (non-stricturing)	72 (13)	62 (93)	
B2 (stricturing)	17 (3)	17 (25)	
B3 (penetrating)	11 (2)	21 (11)	
Montreal classification perianal Crohn’s disease, % (*N*)			0.872
Yes	17 (3)	18 (27)	
No	83 (15)	82 (122)	
Medical treatment at baseline, % (*N*)			
5-acetylsalicylic acid	45 (14)	45 (124)	0.966
Corticosteroids	84 (26)	80 (222)	0.619
TNF-Inhibitors	10 (3)	7 (20)	0.622
Other IBD treatment	3 (1)	1 (4)	0.414
Surgery for IBD before diagnosis, % (*N*)	10 (3)	8 (23)	0.735

IBD, inflammatory bowel disease; TNF, tumour necrosis factor.

Azathioprine was used as first-line thiopurine treatment in 88.6% of the patients with IBD whereas 6-mercaptopurine (62.3%) and thioguanine (27.9%) were most used as second-line thiopurine treatment (Table 2). Side effects that led to discontinuation of thiopurine treatment are shown in Table 3.

**Table 2.** Characteristics of thiopurine treatments.

	Azathioprine (AZA), *N* = 278	6-Mercaptopurine, *N* = 63	AZA/allopurinol*, *N* = 7	Thioguanine, *N* = 31	Total, *N* = 379
Median daily dose in mg (25th–75th percentile)	100 (50–150)	50 (50–75)	25 (25–88) (AZA)	11 (7–19)	
Proportion of patients with Crohn’s disease, % (*N*)	54.7 (151)	58.7 (37)	42 (3)	58.1 (18)	55.4
Termination of treatment, % (*N*)	42.1 (117)	65.1 (41)	28.6 (2)	16.1 (5)	43.5 (165)
Reason for termination, % (*N*)					
Side effects	71.8 (84)	63.4 (26)	100 (2)	20.0 (1)	68.1 (113)
Lack of effect	8.5 (10)	9.8 (4)	0.0 (0)	40.0 (2)	9.6 (16)
Patient’s request	15.4 (18)	22.0 (9)	0.0 (0)	20.0 (1)	17.5 (28)
Other	4.3 (5)	4.9 (2)	0.0 (0)	20.0 (1)	4.8 (8)
Thiopurine treatment attempts, % (*N*)					
1	88.6 (272)	7.8 (24)	1.6 (5)	2.0 (6)	100 (307)
2	9.8 (6)	62.3 (38)	0.0 (0)	27.9 (17)	100 (61)
3	0.0 (0)	10.0 (1)	20.0 (2)	70.0 (7)	100 (10)
4	0.0 (0)	0.0 (0)	0.0 (0)	100 (1)	100 (1)
Total	73.4 (278)	16.6 (63)	1.8 (7)	8.2 (31)	100 (379)

* Azathioprine and allopurinol combination treatment.

**Table 3.** Side effects that led to discontinuation of thiopurine treatment.

	Azathioprine (AZA)	6-Mercaptopurine	AZA/allopurinol*	Thioguanine
First-line treatment				
Proportion of patients that stopped due to side effects, % (*n*)	30.1 (82)	45.8 (11)	40.0 (2)	0 (0)
Side effects that led to discontinuation (*n*)				
Gastrointestinal symptoms	40	4	1	0
Elevated liver enzymes	21	4	0	0
Itching	2	0	0	0
Bone marrow suppression	6	3	0	0
Pancreatitis	6	1	0	0
Fever, elevation of inflammatory markers	9	0	2	0
Joint pain	4	1	1	0
Fatigue	4	0	0	0
Headache	1	0	0	0
Alopecia	1	0	0	0
Angioedema	0	1	0	0
Recurrent infections	1	0	0	0
Unspecific cause	2	1	0	0
Second-line treatment				
Proportion of patients that stopped due to side effects, % (*n*)	33.3 (2)	39.5 (15)	0 (0)	4 (1)
Side effects that led to discontinuation (*n*)				
Gastrointestinal symptoms	0	9	0	0
Elevated liver enzymes	0	3	0	0
Bone marrow suppression	1	0	0	0
Pancreatitis	0	1	0	0
Fever, elevation of inflammatory markers	0	2	0	0
Joint pain	1	2	0	1
Fatigue	0	1	0	0
Headache	0	1	0	0

Some patients had multiple side effects that led to discontinuation of thiopurine treatment. Second-line treatment includes second, third and fourth attempts of thiopurines.

* Azathioprine and allopurinol combination treatment.

### Drug-survival

**Figure 2.**
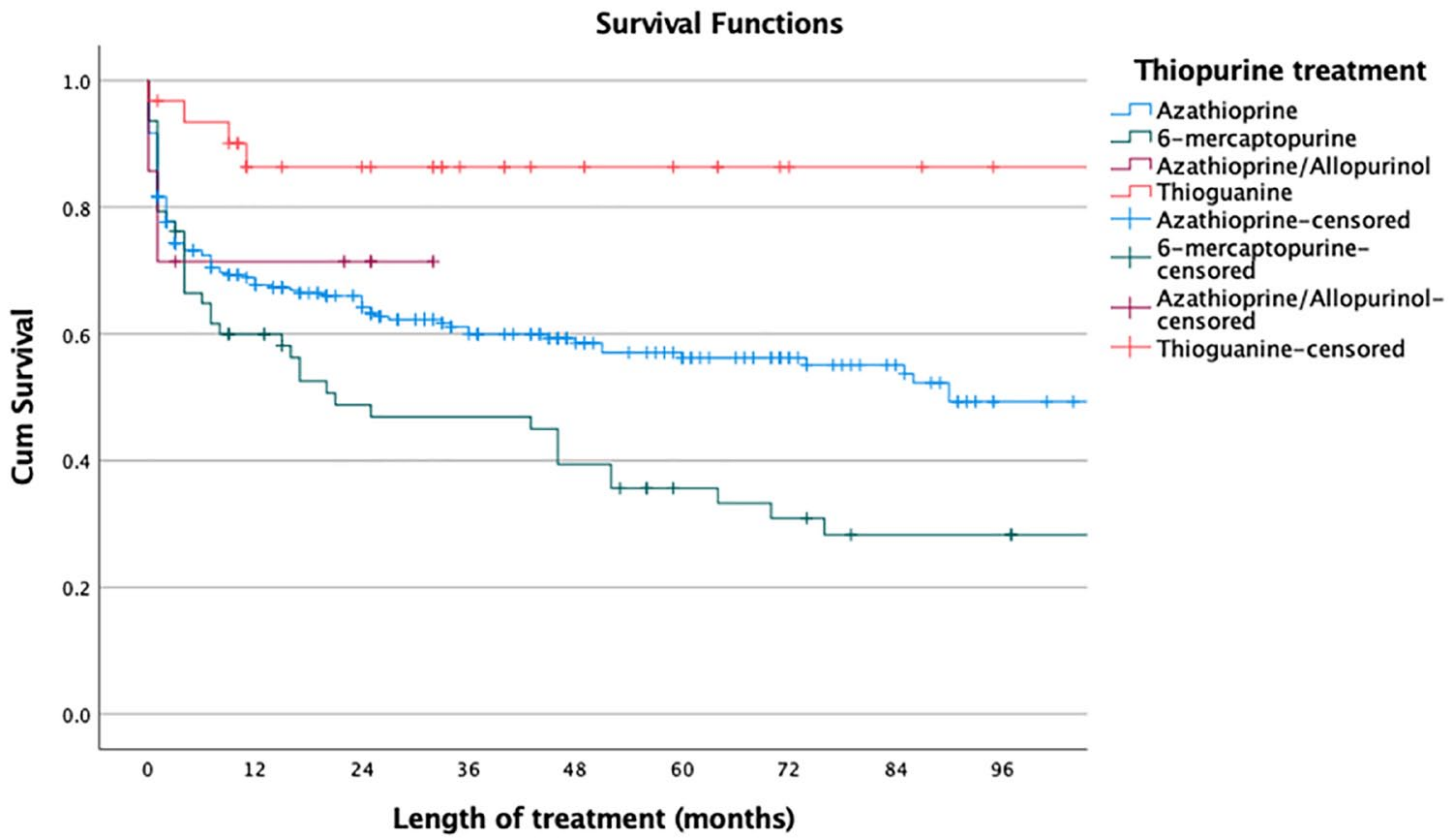
Drug-survival for treatment with azathioprine (*n* = 278), 6-mercaptopurine (*n* = 63), azathioprine/allopurinol (*n* = 7) and thioguanine (*n* = 31) in patients with inflammatory bowel disease. All treatment attempts are included.

**Figure 3.**
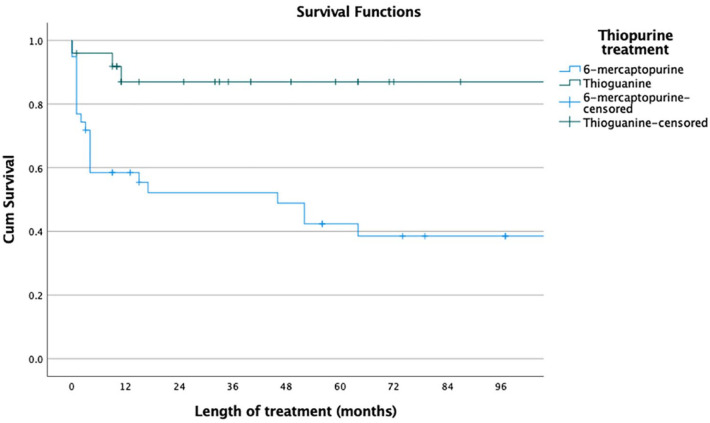
Drug-survival for second-line thiopurine treatment in patients with inflammatory bowel disease. A comparison between patients who received 6-mercaptopurine (*n* = 39) and patients who received thioguanine (*n* = 25).

Overall, when including all thiopurine attempts, the median time on treatment for the different thiopurine treatments were 24 months (25th–75th percentile, 3–59 months) for azathioprine, 15 months (3–59 months) for 6-mecraptopurine, 22 months (1–25 months) for azathioprine/allopurinol and 33 months (11–64 months) for thioguanine. Using the Mantel–Cox rank test, thioguanine had the longest drug-survival (thioguanine *versus* azathioprine *p* = 0.014; thioguanine *versus* 6-mercaptopurine *p* < 0.001); azathioprine had a significantly longer drug-survival than 6-mercaptopurine (*p* = 0.014); and combination therapy with azathioprine and allopurinol had a longer drug-survival than monotherapy with azathioprine (*p* = 0.044) (Figure 2). For second-line thiopurine treatment, the median time on treatment were 13 months (2–69) for 6-mercaptopurine and 33 months (11–64) for thioguanine. Thioguanine had a significantly longer drug-survival than 6-mercaptopurine (Mantel–Cox rank test: *p* = 0.006) (Figure 3). At 60 months, 86% of the patients who started low-dose thioguanine was still on treatment compared to 42% of the patients who started 6-mercaptopurine (*p* = 0.022) (Figure 3).

### Biomarkers

There were no differences in median 6-TGN levels between different thiopurine treatments in the first 5 years of treatment (Table 4). Patients on thioguanine treatment had significantly lower MeTIMP levels (median of the maximum value during the first 5 years) than patients on azathioprine treatment.

**Table 4.** Biochemical markers in the five 5 years after start of thiopurine treatment.

	Azathioprine (AZA), *N* = 278	6-Mercaptopurine, *N* = 63	AZA/allopurinol, *N* = 7	Thioguanine, *N* = 31	All thiopurines, *N* = 379
Median time on treatment (months) (25th–75th percentile)	24 (3–59)	15 (3–58)	22 (2–25)	33 (11–62)	24 (3–59)
Median 6-TGN (25th–75th percentile) (*N* = 127)	335 (234–433)	341 (251–396)	269 (247–463)	364 (303–437)	337 (256–433)
Maximum level of MeTIMP (25th–75th percentile) (*N* = 94)	0.7 (0.4–1.9)	1.6 (0.5–2.3)	0.2 (0.2–1.7)	0.2 (0.2–0.2)*	0.7 (0.3–1.9)
Proportion of patients with low neutrophiles (×10^9^/L) at least once (*N* = 309)	% (*N*)	% (*N*)	% (*N*)	% (*N*)	% (*N*)
⩽1.5	13.8 (32)	21.6 (11)	0.0 (0)	17.4 (4)	15.2 (47)
⩽1.0	2.1 (5)	9.8 (5)	0.0 (0)	0.0 (0)	3.2 (10)
Proportions of patients with an abnormal liver test at least once					
ASAT; µkat/L (*N* = 369)	% (*N*)	% (*N*)	% (*N*)	% (*N*)	% (*N*)
Men ⩾1.5	15.1 (24)	3.0 (1)	20 (1)	12.5 (2)	13.1 (28)
Women ⩾1.2	16.4 (19)	20.8 (5)	0.0 (0)	14.3 (0)	16.7 (26)
Total	1*6.7* (43)	10.5 (6)	14.3 (1)	13.3 (4)	14.6 (54)
ALAT; µkat/L (*N* = 370)	% (*N*)	% (*N*)	% (*N*)	% (*N*)	% (*N*)
Men ⩾2.2	11.9 (19)	14.7 (5)	14.3 (1)	6.3 (1)	12.1% (26)
Women ⩾1.5	18.1 (21)	33.3 (8)	0.0 (0)	57.1 (8)*	22.1 (37)
Total	14.5 (40)	22.4 (13)	14.3 (1)	30.0 (9)	15.9 (63)
ALP; µkat/L (*N* = 369)	% (*N*)	% (*N*)	% (*N*)	% (*N*)	% (*N*)
⩾2.9	10.9 (30)	5.2 (8)	0.0 (0)	16.7 (5)	10.3 (43)
Pancreatic amylase; µkat/L (*N* = 260)	% (*N*)	% (*N*)	% (*N*)	% (*N*)	% (*N*)
⩾2.0	3.4 (7)	4.2 (1)	0.0 (0)	0.0 (0)	3.1 (8)

* Statistically significant (*p* < 0.05) (azathioprine as reference).

ALAT, alanine amino transferase; ALP, alkaline phosphatase; ASAT, aspartate amino transferase.

There were no differences in the proportion of patients that showed abnormal levels of white blood cell counts, ASAT, ALP and pancreatic amylase between the different thiopurine treatments at least once in the first 5 years of treatment. Women with thioguanine treatment significantly more often at least once exceeded ALAT (>1.2 µkat/L) than patients on azathioprine treatment. Only one patient with low-dose thioguanine stopped treatment due to side effects (joint pain).

**Figure 4.**
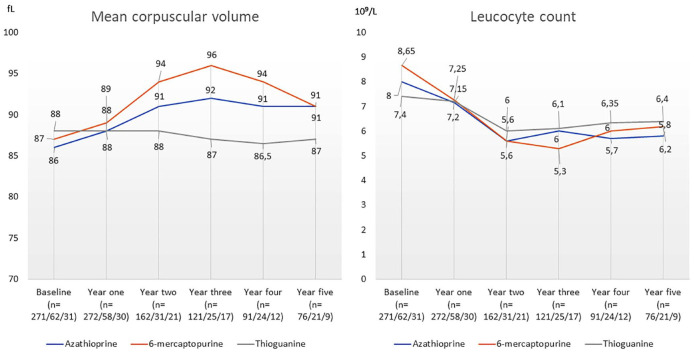
The yearly median mean corpuscular volume and leucocyte count in patients with inflammatory bowel disease who were treated with azathioprine, 6-meraptopurine and thioguanine.

Patients on thioguanine treatment showed significantly lower values of median MCV than patients with azathioprine (Year 2: *p* = 0.004; Year 3: *p* < 0.001; Year 4: *p* = 0.003; Year 5: *p* = 0.013) and patients with 6-mercaptopurine (Year 1: *p* = 0.033; Year 2: *p* < 0.001; Year 3: *p* < 0.001; Year 4: *p* < 0.001; Year 5: *p* = 0.004) (Figure 4). Patients on 6-mercaptopurine treatment had significantly higher levels of MCV than patients on azathioprine treatment (Year 1: *p* = 0.028; Year 2: *p* = 0.012; Year 3: *p* = 0.005; Year 4: *p* = 0.044). There were no differences in median leucocyte levels between patients treated with azathioprine, 6-mercaptopurin and thioguanine (Figure 4).

### Inflammatory activity

The proportion of patients treated with TNF-inhibitors was significantly higher the first year for patients treated with thioguanine than patients treated with azathioprine (42% *versus* 21%; *p* = 0.008), but after the first year, there was no differences between the different thiopurine groups (Table 5). Patients treated with thioguanine had significantly lower serum levels of albumin than patient treated with azathioprine the first 2 years. Patients treated with 6-mercaptopurine had significantly lower faecal calprotectin levels than patients treated with azathioprine during year 3 and 4 after start of thiopurine treatment (Tables 5 and 6).

**Table 5.** The proportion of patients with combination therapy with TNF-inhibitors and inflammatory markers 5 years after start of thiopurine treatment.

	Baseline (*N* = 372)	Year 1 (*N* = 371)	Year 2 (*N* = 227)	Year 3 (*N* = 182)	Year 4 (*N* = 145)	Year 5 (*N* = 118)
Proportion of patients with combination therapy, % (*N*)						
Azathioprine	6.8 (19)	20.9 (58)	29.0 (49)	37.8 (51)	38.1 (40)	36.9 (31)
6-mercaptopurine	1.6 (1)	20.6 (13)	33.3 (12)	35.7 (10)	29.6 (8)	32.0 (8)
Thioguanine	9.7 (3)	41.9 (13)*	40.9 (9)	31.6 (6)	38.5 (5)	33.3 (4)
Median faecal calprotectin, µg/g						
Azathioprine	598	202	94	85	160	132
6-Mercaptopurine	581	170	118	59*	28*	55
Thioguanine	595	364	293	88	96	72
Proportion of patients with annual median faecal calprotectin <200 µg/g, % (*N*)						
Azathioprine	23.5 (60)	50.0 (97)	63.8 (87)	65.6 (63)	54.2 (39)	66.1 (39)
6-Mercaptopurine	22.8 (13)	52.4 (22)	61.9 (13)	78.9 (15)	76.2 (15)	66.7 (10)
Thioguanine	13.8 (4)	37.5 (9)	47.6 (10)	63.6 (7)	63.6 (7)	71.4 (5)
Median C-reactive protein						
Azathioprine	5.00	3.00	1.20	1.00	1.00	0.95
6-Mercaptopurine	4.85	4.80	1.30	1.00	0.90	1.30
Thioguanine	2.95	2.40	2.20	1.90	1.60	2.40
Median albumin, g/L						
Azathioprine	38.0	40.0	40.0	40.0	41.0	40.6
6-Mercaptopurine	39.0	41.0	41.0	41.0	39.5	40.0
Thioguanine	38.0	37.0*	39.0*	39.5	38.5	38.0
Median haemoglobin, g/L						
Azathioprine	134.5	134.0	136.5	138.5	139.0	139.0
6-Mercaptopurine	135.0	134.0	135.0	134.0	134.5	138.0
Thioguanine	136.0	131.0	133.0	138.0	135.0	133.0

* Statistically significant (*p* < 0.05) with azathioprine as reference.

TNF, tumour necrosis factor.

**Table 6.** Inflammatory markers in patients on thiopurine monotherapy, the first 5 years of treatment.

	Year 1 (*N* = 248)	Year 2 (*N* = 113)	Year 3 (*N* = 77)	Year 4 (*N* = 65)	Year 5 (*N* = 51)
Median faecal calprotectin, µg/g					
Azathioprine	159	92	109	201	139
6-Mercaptopurine	188	269	59*	25*	55
Thioguanine	232	293	50	87	48
Proportion of patients with annual median faecal calprotectin <200 µg/g, % (*N*)					
Azathioprine	57.7(82)	64.4 (56)	60.7 (34)	48.9 (22)	65.0 (26)
6-Mercaptopurine	51.5 (17)	46.2 (6)	83.3 (10)	84.6 (11)*	71.4 (5)
Thioguanine	38.5 (5)	46.2 (6)	66.7 (6)	71.4 (5)	66.7 (3)
Median C-reactive protein					
Azathioprine	3.25	1.40	1.40	1.60	1.10
6-Mercaptopurine	5.00	1.20	1.20	1.45	2.00
Thioguanine	2.00	2.40	1.55	0.95	1.90

* Statistically significant (*p* < 0.05) with azathioprine as reference.

## Discussion

This retrospective observational study showed, when including all attempts with thiopurine treatment, that low-dose thioguanine treatment had significantly better drug-survival than both azathioprine treatment and 6-mercaptopurine treatment despite that the patient failed or were intolerant to previous thiopurine treatment. When comparing patients who failed or were intolerant to azathioprine treatment, thioguanine showed significantly better drug-survival than 6-mercaptopurine. Furthermore, in patients who started second-line therapy, 86% of the patients with low-dose thioguanine were still on treatment at 60 months. The drug-survival of low-dose thioguanine in our study was higher than reported in a previous study from the United Kingdom that used a higher dose of thioguanine.^19^ In that study, approximately 55% of the patients on a dose of 20 mg/day and approximately 47% on a dose of 40 mg/day was still on treatment at 60 months.

In daily doses of 40 mg of thioguanine, 23–56% of patients had to terminate treatment due to intolerance.^20–23^ But in lower doses of thioguanine, there are in general relatively few adverse events associated with thioguanine use in patients with IBD.^9^ For example, using daily doses of 20 mg or lower, the proportion of patients that terminated thioguanine treatment due to adverse events was lower (10–20%) despite that most of the patients in those studies were intolerant to previous thiopurine treatment.^14,24–27^. Interestingly, the clinical response rate of a low dose of thioguanine (lower than 20 mg) was similar in a meta-analysis to that of a higher dose of thioguanine (more than 20 mg).^28^

In our study, when using a median dosage of 11 mg of thioguanine only 1 out of 31 patients (6%) terminated treatment due to side effects. The 6-TGN levels were similar in the patients treated with low-dose thioguanine compared to other thiopurine treatments. Consistent with our result, Pavlidis *et al*.^29^ used split-doses of thioguanine therapy of 20 mg once, every second day or every third day, and only 2 out of 62 (3%) patients discontinued treatment due to adverse events. Interestingly 78% of the patients in that study had a clinical response to thioguanine and only 14% did not benefit from treatment. Although our study did not aim to evaluate clinical response, we did find a similar response on inflammatory markers the first 5 years from starting treatment with thioguanines compared to conventional thiopurines.

Thioguanine has been associated with nodular regenerative hyperplasia (NRH) in the liver which has been found in 4% of the patients treated with thioguanine^28^ In the present study, we had no information on the presence of NRH in the liver. In a recently published meta-analysis, the risk for NRH increases with dosages and the authors concluded that the low dose of thioguanine may be an efficacious therapy with lower risk for cytopenia and NRH.^28^

It was suggested that the MCV correlates with erythrocyte 6-TGN concentrations in patients treated with thiopurines.^30^ For example, in treatment with azathioprine or in combination treatment with azathioprine and infliximab, a higher MCV was associated with mucosal healing in patients with CD.^31^

In our study, the patients treated with low-dose thioguanine showed a significantly lower MCV than patients treated with azathioprine and 6-mercaptopurine. In the literature, there is little data on how thioguanine affects the MCV. Meijer *et al.*^32^ studied complete blood count outcomes in patients with IBD treated with different thiopurines. In patients treated with conventional thiopurines these previous authors found that 6-TGN correlated negatively with haemoglobin concentrations and white blood cell counts and correlated positively with MCV. However, in patients treated with thioguanine, there was no such correlations which indicate that thioguanine does not affect bone marrow function to the same extent as conventional thiopurines.

There is uncertainty regarding what 6-TGN level is the most optimal for thioguanine treatment. A higher level (>700 pmol/8 × 10^8^) than that recommended for conventional thiopurines (235–450 pmol/8 × 10^8^)^11^ has been proposed.^33^ In our study, the median 6-TGN level in the patients treated with low-dose thioguanine was 346 pmol/8 × 10^8^.

The present study has several limitations. Firstly, the number of patients that was treated with thioguanine in our study was small. Secondly, the study is based on retrospective clinical data and could be biased by what doctors prefer in treatment choice, patient characteristics, and we lack detailed data on other concomitant drugs for IBD. Thirdly, we have no data on clinical effect except for yearly median levels of inflammatory biomarkers. The clinical effects in the long term is difficult to access in observational studies due to concomitant treatments and different patient characteristics. The strength of the study is the long follow-up time, and we present drug-survival data on thioguanine in relation to conventional thiopurine treatment.

To conclude, a low dose of thioguanine is well-tolerated in patients with IBD and have a significantly higher drug-survival than conventional thiopurines. There is a need for prospective studies that evaluate clinical effects with different doses of thioguanine in comparison to placebo or other drugs for IBD.

## Supplemental Material

sj-docx-1-tag-10.1177_17562848241228064 – Supplemental material for The drug-survival of low-dose thioguanine in patients with inflammatory bowel disease: a retrospective observational study Supplemental material, sj-docx-1-tag-10.1177_17562848241228064 for The drug-survival of low-dose thioguanine in patients with inflammatory bowel disease: a retrospective observational study by Helena Gensmyr-Singer, Mårten Werner and Pontus Karling in Therapeutic Advances in Gastroenterology

## Declarations

**Ethics approval and consent to participate:** The study was judged by the National Ethical Review Board (Dnr 2022-060609-01) and the board had no ethical concerns. The study was performed in accordance with the Swedish law for Patient Safety and directions of the Swedish Data Protection Authority. Due to the retrospective observational design of the study and that the study evaluated clinical practice there was no consent obtained from the patients.

**Consent for publication:** All authors have given consent for publication.

**Availability of data and materials:** Data are available from the corresponding author on reasonable request.
